# 437. Longitudinal Plasma Cytokine Profiles Differentiating COVID-19 Severity Groups

**DOI:** 10.1093/ofid/ofab466.636

**Published:** 2021-12-04

**Authors:** Amanda M Green, Aisha Souquette, Mona Agrawal, Joshua Wolf, Joshua Wolf, Aditya Gaur, Kim J Allison, Jeremie Estepp, Emma Allen, Paul Thomas, Heather Smallwood

**Affiliations:** 1 St Jude Children’s Research Institute, Memphis, Tennessee; 2 University of Tennessee Health Science Center, Memphis, Tennessee; 3 St. Jude’s Children’s Research Hospital, Memphis, TN; 4 St Jude Children’s Research Hospital, Memphis, Tennessee; 5 St. Jude Children’s Research Hospital, Memphis, TN

## Abstract

**Background:**

Severe acute respiratory syndrome coronavirus 2 (SARS-CoV-2) causes coronavirus disease 2019 (COVID-19), an infection with widely varying clinical severity. Severe COVID-19 was initially proposed to be secondary to cytokine storm syndrome (CSS). However, studies since showed that patients with severe COVID-19 rarely display CSS cytokine phenotypes, and may have more limited inflammatory responses instead.

**Methods:**

Prospective cohorts, aged 0-90 years of age who tested positive by polymerase chain reaction (PCR) for SARS-CoV-2 were enrolled from inpatient hospitals and outpatient testing centers in Memphis, TN from May 2020-January 2021. Longitudinal blood samples were obtained including acute, sub-acute and convalescent timepoints. Severity scores of asymptomatic, mild, moderate, and severe COVID-19 were assigned at time of convalescent assessment. Plasma was analyzed with a quantitative human magnetic 38-plex cytokine assay.

**Results:**

: 169 participants were enrolled, including 8 asymptomatic, 117 mild, 22 moderate and 17 severe cases, and 5 children with post-COVID-19 multisystem inflammatory syndrome in children (MIS-C). All moderate and severe patients were hospitalized and received treatment (39%). Clear distinctions were seen between asymptomatic-mild cases and moderate-severe cases at acute timepoints and during disease progression for GCSF, IL-8, IL-10, IL-15, IL-1Ra, IP-10, MIP-1a, MIP-1β, and TGFα. There was a significant difference between participants who did and did not require hospitalization for acute timepoint levels of IL-10, IL-15, MIP-1 β and TGFα (*p*< 0.01). Only 4 participants with active COVID-19 were found to meet criteria for CSS (2%), only 3 of which were severe. MIS-C participants showed nearly universally elevated cytokine levels compared to those with active COVID-19.

Temporal and severity associations of IL-10 and IP-10

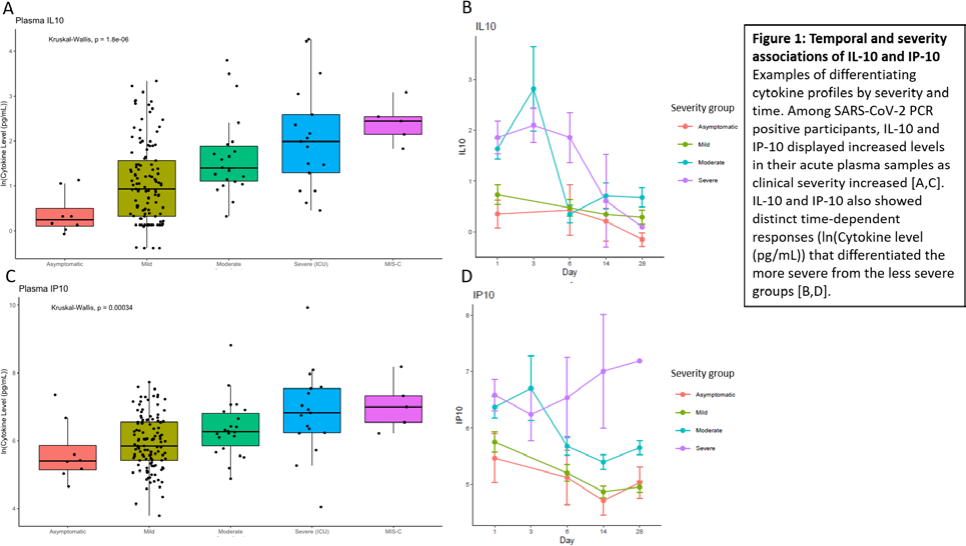

Figure 1. Temporal and severity associations of IL-10 and IP-10 Examples of differentiating cytokine profiles by severity and time. Among SARS-CoV-2 PCR positive participants, IL-10 and IP-10 displayed increased levels in their acute plasma samples as clinical severity increased [A,C]. IL-10 and IP-10 also showed distinct time-dependent responses (ln(Cytokine level (pg/mL)) that differentiated the more severe from the less severe groups [B,D].

**Conclusion:**

Moderate and severe acute COVID-19 has a distinct cytokine profile from asymptomatic and mild cases, as detected from acute, subacute and convalescent plasma.

**Disclosures:**

**Joshua Wolf, MBBS, PhD, FRACP**, **Karius Inc.** (Research Grant or Support) **Joshua Wolf, MBBS, PhD, FRACP**, Nothing to disclose **Paul Thomas, PhD**, **Cytoagents** (Consultant)**Immunoscape** (Consultant)

